# Role of suppression of endometriosis with progestins before IVF-ET: a non-inferiority randomized controlled trial

**DOI:** 10.1186/s12884-021-03736-2

**Published:** 2021-03-30

**Authors:** Eissa Khalifa, Hashem Mohammad, Ameer Abdullah, Mazen Abdel-Rasheed, Mohammed Khairy, Mahmoud Hosni

**Affiliations:** 1 Obstetrics and Gynecology Department, Faculty of Medicine, Minia University, Minia, Egypt; 2 Reproductive Health Research Department, National Research Centre, 33 El-Buhouth St, Dokki, Cairo, 12622 Egypt

**Keywords:** Endometriosis, IVF-ICSI, Dienogest, Progestins, GnRH-analogue

## Abstract

**Background:**

Endometriosis affects the responsiveness to ovarian stimulation. This study aimed to assess the role of Dienogest pretreatment for endometriosis suppression as compared to Gonadotropin-releasing hormone agonist (GnRHa) in patients with endometriosis pursuing IVF treatment.

**Methods:**

In this randomized controlled trial, 134 women with endometriosis-related infertility were randomly allocated to group A (*n* = 67) who had monthly depot GnRHa for 3 months before ovarian stimulation in IVF treatment (Ultra-long protocol), and Group B (*n* = 67) who had daily oral Dienogest 2 mg/d for 3 months before starting standard long protocol for IVF. The primary outcome measure was the number of oocytes retrieved. The secondary outcome measures included the number of mature oocytes, fertilization rate, quality of life assessed by FertiQoL scores, cost of treatment, and pregnancy outcomes.

**Results:**

Although there was no statistically significant difference between both groups regarding ovarian stimulation, response parameters, and pregnancy outcomes, the Dienogest group had a lower cost of treatment (2773 vs. 3664 EGP, *P* < 0.001), lower side effects (29.9% vs. 59.7%, *P* < 0.001), higher FertiQoL treatment scores (33.2 vs. 25.1, *P* < 0.001) and higher tolerability scores (14.1 vs. 9.4, *P* < 0.001 < 0.001).

**Conclusion:**

Our study indicates that Dienogest is a suitable and safe substitute for GnRHa pretreatment in endometriosis patients.

**Trial registration:**

NCT04500743 “Retrospectively registered on August 5, 2020”.

## Background

Endometriosis affects nearly 15% of patients with infertility requiring assisted reproduction treatment. It is believed that endometriotic lesions in the pelvis create a hostile microenvironment for the fertilization of oocytes and the early development of an embryo in the fallopian tubes in vivo. Ovarian endometriosis also may affect the ovarian reserve and responsiveness to ovarian stimulation in IVF programs. These effects lead to lower fertilization and pregnancy rates in patients with endometriosis pursuing IVF compared with other groups of patients [1]. This is probably related to an increased level of pro-inflammatory cytokines and abnormal oxidation damage to ovarian follicles, resulting in diminished oocyte quality regardless of endometriosis stage [2–4].

Continuous GnRH agonist (GnRH-a) administration causes a down-regulation of GnRH-a receptors on pituitary gonadotropins and a severe hypo-estrogenic state, causing suppression of endometriotic lesions. This has led to the use of long-acting depot GnRH-a preparation for control of pelvic pain and other endometriosis-related symptoms [5]. Its use alone without adding back hormonal therapy should be to a maximum of 6 months as long-term use of GnRH-a is associated with hypo-estrogenic adverse effects, such as hot flushes, vaginal dryness, decreased libido, and decreased bone mineral density [6].

In the same vein, it has been proposed that long-term GnRH-a pretreatment for 3 months should be used in patients with endometriosis before IVF treatment to suppress any endometriotic lesions [7]. This so-called ultra-long protocol has been suggested to improve the pregnancy rates in endometriosis patients after IVF compared with other protocols for pituitary down-regulation. However, systematic reviews have shown conflicting evidence [8–10]. The relatively long period of hypo-estrogenic side effects associated with an ultra-long protocol with a potential need for higher doses of gonadotropins and recent evidence from Cochrane review showing inconclusive evidence of benefit has led to research into better alternatives for control of endometriosis before IVF [9].

Dienogest (DNG) is a fourth-generation selective progestin (highly selective for binding to progesterone receptors in endometrial tissue). It has a potent oral progesterone activity, a little androgenic or estrogenic activity. It reduces the activity of endometriotic lesions by creating a local dominant progesterone effect while suppressing the estrogen effect moderately, and it is thought to have anti-angiogenic and anti-inflammatory effects [11, 12]. The incidence of hypo-estrogenic side effects with Dienogest is not prominent. This has led to the use of Dienogest for control of pelvic pain due to endometriosis with beneficial and comparable effect to depot GnRHa [11, 13–15]. However, its role in suppressing the endometriotic lesions before IVF treatment and its impact on IVF outcome is still unclear [16, 17].

The study aimed to compare the impact of pretreatment with Dienogest for 3 months in women diagnosed with endometriosis and attending for IVF with pretreatment with GnRH-a similar patients group regarding ovarian response, pregnancy outcomes, and quality of life.

## Methods

### Study design and participants

This was a parallel-group open-label randomized controlled clinical trial conducted at Minia Infertility Research Unit (MIRU) in Egypt during the period from 8/2018 until 10/2019. All consecutive patients attending for IVF at MIRU were assessed for eligibility for inclusion in the study. Women were deemed eligible for inclusion if they had a confirmed diagnosis of endometriosis by laparoscopy in the last 2 years, their age at the start of treatment < 40 years, and their body mass index < 35 Kg/m2. Women were excluded if they have been already on long-term down-regulation of the pituitary gland with GnRHa for control of endometriosis or if they have liver or kidney disease precluding the use of Dienogest or have evidence of diminished ovarian reserve (e.g., high FSH level > 12 IU/L, low AMH level < 1.1 ng/ml or low antral follicle number < 7). All patients were allowed to participate once in the trial with no second entry after a failed IVF cycle.

The study was ethically approved by the institutional review board of the Faculty of Medicine, Minia University, and was registered at an international clinical trial registry (NCT04500743). All eligible women were given verbal and written information about the trial, and written informed consents were obtained before enrollment. Randomization was done using computer-generated random sequence numbers generated by the clinical research unit at the Faculty of Medicine, Minia University, with concealment of allocation by sealed opaque envelopes. Due to the nature of the intervention and its design, it was impossible to blind participants or clinicians. However, physicians who performed the oocyte retrieval and the embryo transfer were blinded to the group type to minimize bias. All participants were followed by the clinical research team at MIRU till 12 weeks pregnant.

### Study protocol

**Fig. 1:**
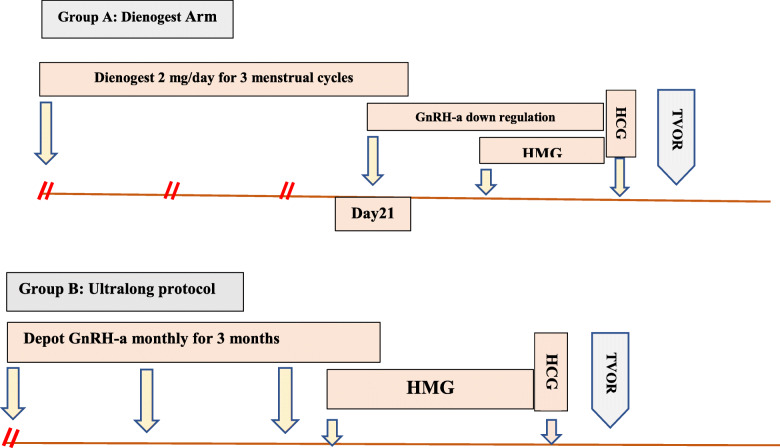
Flowchart of ovarian stimulation in both groups

All patients had workup before starting IVF treatment, including pelvic ultrasound assessment of the antral follicle count (AFC), measurements of Anti-Mullerian Hormone (AMH), and Follicle-Stimulating Hormone (FSH), and basic semen analysis. All eligible patients were randomly assigned at the point of enrollment into two groups;

1. Group A included patients who had pretreatment with GnRH-a in the form of depot leuprorelin acetate (LEUCRAN, ABVIA, EGYPT) 3.75 mg SC monthly injections for 3 months before starting ovarian stimulation after the third dose of depot GnRH-a., and
2. Group B included women who had pretreatment with Dienogest 2 mg tablet orally daily for 3 months. In the last 3 weeks of the pretreatment period, short-acting GnRH-a was started in a dose of 0.5 mg SC daily followed by ovarian stimulation when Dienogest was stopped, as shown in Fig. 1.

The ovarian stimulation was started in both groups with a flexible dose of Human menopausal gonadotropin (HMG, Fostimon® or Merional®, IBSA, EGYPT) modified according to the patients’ age, FSH level, total antral follicle count at the start of ovarian stimulation, polycystic ovary status, and previous ovarian response. In brief, patients under 35 years were given 150 IU/day, and patients 35–40 years were given 225 IU/day. The ovarian stimulation dose was increased by 75 IU/day for corresponding age groups if they had a suboptimal ovarian response in a previous cycle of (4–7) oocytes or have BMI > 30 Kg/ m2. Patients were monitored for the ovarian response with serial transvaginal ultrasound scans starting from day 8 of ovarian stimulation until there were at least three follicles > 17 mm in average diameter when Human Chorionic Gonadotropin (HCG) was administered in a dose of 10,000 IU IM (Profasi/Pregnyl). After that, patients were booked for transvaginal oocyte retrieval under general anesthesia 36 h later. During monitoring, further step-down or step-up of the dose was done according to the patient’s response.

Fertilization was achieved by either conventional IVF or ICSI, depending mainly on the sperm parameters on the day of oocyte retrieval, duration of subfertility, and previous IVF performance. Embryo transfer was done under ultrasound guidance on day 3 or 5, depending on the number and quality of the developing cohort of embryos. Patients were given progesterone vaginal suppositories (Uterogestan or Cyclogest 400 mg PV BD) starting on the oocyte retrieval day and continued for 16 days until the day of the urinary pregnancy test. All patients enrolled in the study were planned to have a fresh embryo transfer with no frozen/thawed embryo transfer included in the analysis, and all patients were only allowed to have 1 cycle of treatment.

Data on the participants’ baseline characteristics, their ovarian response, and the pregnancy outcomes were collected prospectively in a dedicated register. At the start of enrollment of the study, patients were given the Arabic version of the FertiQoL questionnaire and asked to answer the questions in the first (core) part of the questionnaire regarding their overall satisfaction and quality of life and the domains on emotional, mind/body, relational and social functioning. At the consultation to confirm pituitary down-regulation before starting ovarian stimulation, patients were asked to answer the questions regarding the treatment part to assess their experience during the pretreatment period. Answers to these questions in the FertiQoL were scored according to the scoring scheme suggested by the Cardiff University group [18]. Patients were also asked verbally about any side effects that they have experienced during the same period.

### Study outcomes

The primary outcome measure of this study was the number of retrieved oocytes, as the main concern was the effect of either GnRHa or Dienogest on ovarian responsiveness. The secondary outcome measures were the fertilization rate (defined as the number of zygotes with two pronuclei divided by the number of oocytes), the number of transferrable embryos (defined as the number of embryos suitable for transfer in the stimulated cycle or cryopreservation), the cost of the treatment in Egyptian pounds including cost of pretreatment and ovarian stimulation drugs, the pregnancy rate per cycle started (defined as patients with positive urinary or serum pregnancy test divided by the number of patients starting the treatment), the clinical pregnancy rate per cycle started (defined as the number of patients with at least one intrauterine gestational sac with identifiable fetal heart pulsations over the total number of patients starting the treatment), and the miscarriage rate (defined as patients with identified intrauterine gestational sac without a fetal pole, or a fetal pole with no heart pulsations with no other viable fetuses over the number of patients with positive pregnancy test). Other secondary outcomes were the patient’s quality of life during the pretreatment period as assessed by the FertiQoL questionnaire and side effects documentation.

### Statistical analysis

This study was powered to assess the difference in the mean number of retrieved oocytes of 2 with a standard deviation of 1.5 (allowing for uncertainty regarding the mean number of retrieved oocytes (range from 5 to 11) and variance (range of standard deviation from 1 to 3) in a parallel independent cohort design with 1:1 ratio of randomization and allowing for type I error of 5% (significance level < 0.05) and type II error of 20% (power of 80%). An estimated sample size of 120 (60 patients in each arm) was deemed suitable, and to allow for a dropout rate of 10%, we aimed to recruit 132 patients.

The groups’ baseline characteristics were tabulated as means ± standard deviation or frequency/percentages. Differences in baseline characteristics and outcomes between groups were evaluated by independent t-test when normally distributed and by Mann-Whitney test when non-normally distributed for continuous variables and chi-square test for categorical ones. A *P*-value < 0.05 was considered significant. All analyses were carried out with SPSS software for Windows version 19.0 (SPSS Inc. Chicago, IL.)

## Results

**Fig. 2:**
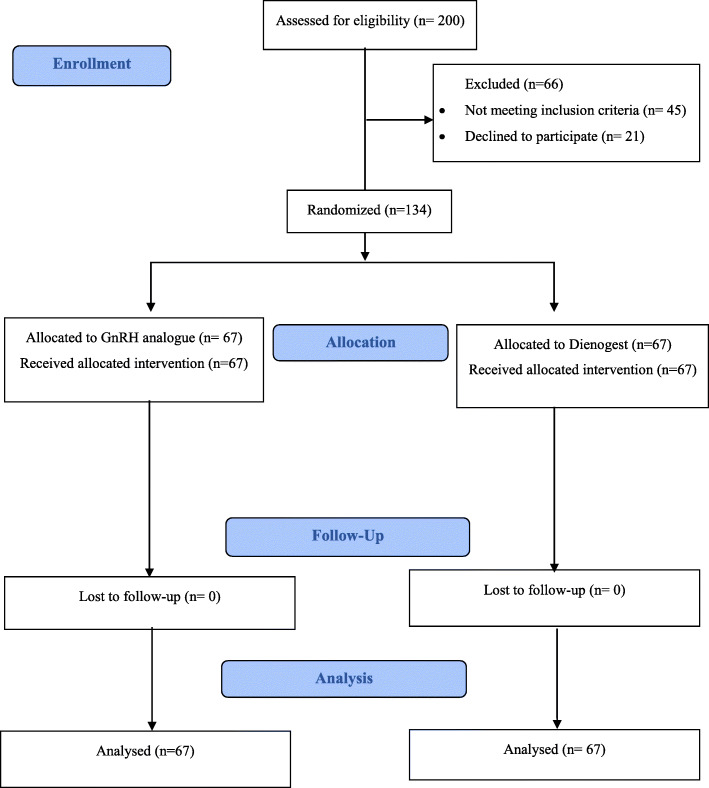
Flowchart of participants in the trial comparing GnRH agonist versus Dienogest pretreatment in endometriosis patients before ART

Following the CONSORT guidelines, 200 patients were assessed for enrollment eligibility in the trial, and 66 patients were excluded, as 45 patients did not meet the eligibility criteria, and 21 patients declined to participate. Patients that were eligible and consented to participate were enrolled in the study, and all patients enrolled completed the trial period with no dropouts, as shown in Fig. 2. The participants’ baseline characteristics (age, body mass index, duration of subfertility, causes of subfertility, grades of endometriosis, baseline FSH, and anti-Mullerian hormone) did not differ significantly between the two intervention groups, as shown in Table 1.

**Table 1:** Baseline characteristics (mean/SD) of the two groups of endometriotic patients having GnRH agonist or Dienogest as a pretreatment before ART

	GnRH agonist	Dienogest	***P***-value
Age (years)^a^	35.6 ± 3.5	36.1 ± 2.7	0.24
BMI (Kg/m2)^a^	22.5 ± 1.6	22.3 ± 1.9	0.18
Duration of subfertility^a^	6.6 ± 1.5	7.2 ± 1.7	0.1
Previous ART (%) ^b^	20/67 (29.85%)	17/67 (25.37%)	0.56
Previous live birth (%) ^b^	10/67 (14.93%)	10/67 (14.93%)	1
Cause of infertility (%) ^b^			
Male	25 (37.31%)	27 (40.3%)	–
Anovulation	27 (40.3%)	30 (44.78%)	
Tubal	32 (47.76%)	32 (47.76%)	
uterine	23 (34.33%)	30 (44.78%)	
Stage of Endometriosis ^b^			
Minimal	5/67 (7.46%)	8/67 (11.94%)	0.69
Mild	13/67 (19.4%)	12/67 (17.91%)	
Moderate	27/67 (40.3%)	22/67 (32.84%)	
Severe	22/67 (32.84%)	25/67 (37.31%)	
FSH (IU/L)^a^	5.4 ± 1.7	4.3 ± 1.8	0.22
AMH (ng/ml)^a^	3.5 ± 1.3	2.8 ± 1.8	0.07
AFC^a^	12 ± 3.2	11.8 ± 2.3	0.08

*FSH* Follicle-stimulating Hormone, *AMH* Anti-Mullerian Hormone, *AFC* Antral Follicle Count

^a^Values are expressed as means ± standard deviations

^b^Values expressed as percentages

There was also no difference regarding the severity of endometriosis between the two groups. There was also no statistically significant difference between both groups regarding ovarian stimulation and response parameters (total dose of FSH injections required, number of oocytes retrieved, number of metaphase II oocytes, fertilization rate), and pregnancy outcomes (pregnancy rate, clinical pregnancy rate, miscarriage rate). However, there was a statistically significant difference between the mean combined cost of pretreatment with GnRH agonist and ovarian stimulation (3664 LE), and the mean combined cost of Dienogest pretreatment and ovarian stimulation (2773 LE) with *p*-value < 0.001 (Table 2).

**Table 2:** Outcomes of assisted reproduction treatment cycles after pretreatment with either GnRH agonist or Dienogest

Outcome	GnRH agonist	Dienogest	***P***-value
Total dose of FSH (IU)^a^	2047 ± 67.7	2180 ± 57.4	0.65
No. of oocyte^a^	11.4 ± 1.2	11.1 ± 1.4	0.78
No. of mature oocytes^a^	6.6 ± 1.3	6 ± 1.8	0.71
Fertilization rate (%) ^b^	40.3%	47.67%	0.38
No. of transferrable embryos^a^	4.5 ± 1.8	5.1 ± 2.1	0.63
Transferred embryo ^b^			
Cleavage stage	37 (55.22%)	42 (62.69%)	0.37
Blastocyst stage	30 (44.78%)	25 (37.31%)	
Pregnancy rate (%) ^b^	15/67 (22.39%)	1 7/67 (25.37%)	0.69
Clinical pregnancy rate (%) ^b^	12/67 (17.91%)	17/67 (25.37%)	0.29
Miscarriage rate (%) ^b^	2/15 (13.3%)	0	–
Cost of pretreatment /ovarian stimulation (EGP)^a^	3664 ± 45.1	2773 ± 38.1	< 0.001

*EGP* Egyptian Pounds, *GnRHa* Gonadotropin-releasing hormone agonist, *FSH* Follicle-stimulating Hormone

^a^Values are expressed as means ± standard deviations

^b^Values expressed as numbers and percentages

The quality of life of patients was assessed by the FertiQoL questionnaire (Table 3), which was administered before the pretreatment period (core part) and before the start of the ovarian stimulation (treatment part). Neither the overall rating of the quality of life nor the core FertiQoL score with its four subsets of emotional, mind/body, relational nor social domains differs significantly at the start of treatment between the two groups. However, there was a statistically significant difference between the two groups, with the overall FertiQoL treatment and tolerability scores favoring the Dienogest treatment. There were no major or life-threatening side effects reported in either group requiring stop or withdrawal from both treatment groups. There were 40 patients (59.7%) in the GnRHa group reporting side effects of hot flushes (*n* = 11), body aches(*n* = 6), sleep disturbances (*n* = 8), low mood (*n* = 8), vaginal dryness (*n* = 7) and in the Dienogest group a total of 20 patients (29.9%) reporting side effects of headache (*n* = 13), breast pain (*n* = 7). The difference in the number of patients reporting these minor side effects was statistically significant, favoring lower side effects profile in the Dienogest group.

**Table 3:** Quality of life assessed by the FertiQoL questionnaire and side effects of GnRHa and Dienogest pretreatment protocols before ART for endometriotic patients

	GnRH agonist	Dienogest	***P***-value
Total FertiQoL score ^a^	94.2 ± 11.5	105 ± 12.6	0.04
Overall quality of life ^a^	2.9 ± 1.1	3.1 ± 0.7	0.78
Overall physical health satisfaction ^a^	3.2 ± 0.6	3.1 ± 0.3	0.82
FertiQoL Core score ^a^	71.8 ± 12.5	72.1 ± 13.5	0.67
Emotional score	18.6 ± 8.3	18.2 ± 7.9	0.54
Mind/Body score	17.4 ± 8.2	19.4 ± 8.8	0.77
Relational score	18.9 ± 7.6	18.4 ± 7.3	0.67
Social score	17.1 ± 6.2	17.5 ± 5.2	0.80
FertiQoL Treatment score ^a^	25.1 ± 7.8	33.2 ± 6.2	< 0.001
Tolerability	9.4 ± 2.3	14.1 ± 3.1	< 0.001
Environment	16.6 ± 4.4	18.2 ± 3.7	0.15
Side effects ^b^	40/67 (59.7%)	20/67 (29.9%)	< 0.001

Side effects in the GnRH agonist included; hot flushes (*n* = 11), body aches (*n* = 6), sleep disturbances (*n* = 8), low mood (*n* = 8) and vaginal dryness (*n* = 7)

Side effects in the Dienogest included; headache (*n* = 13) and Breast pain (*n* = 7)

^a^Values are expressed as means ± standard deviations

^b^Values expressed as numbers and percentages

## Discussion

For the last two decades, the ultra-long protocol of pituitary down-regulation using GnRH agonist has been promoted as the recommended treatment protocol for patients with endometriosis undergoing ART. The presumed benefits of the ultra-long protocol are maintained suppression of endometriotic implants with improvement in clinical and ongoing pregnancy rates [7, 19]. This has recently been proposed also to improve the pregnancy rate in patients with associated adenomyosis [11]. The disadvantages of the ultra-long protocol compared to the standard long protocol are the longer period of pituitary/ovarian suppression with a higher reported rate of hypo-estrogenic side effects and lower ovarian responsiveness. It should be noted that a recent systematic review has not shown a significant benefit of the ultralong protocol on live birth outcomes for patients with endometriosis undergoing IVF [9]. This, however, contradicts another systematic review demonstrating a beneficial effect of the ultralong protocol over long/antagonist protocols in the same group of patients [8].

Dienogest, a synthetic progestin, has been extensively studied recently to manage pelvic pain in endometriosis. In a recent systematic review of 9 randomized controlled trials, Dienogest in a dose of 2 mg/day for periods ranging from 3 to 12 months was more effective than placebo and was comparable to GnRH agonist in controlling endometriosis symptoms [11]. However, like other medical treatments for suppressing endometriosis, Dienogest is not suitable for patients trying naturally for pregnancy because of its central effect of pituitary suppression. With its endometriotic suppressive effect, this latter effect places it as an ideal substitute for the use of GnRHa as a pretreatment before ART treatment. Moreover, the side effects profile of Dienogest reported from RCTs was also minimal.

This study has shown that suppressing endometriosis before ovarian stimulation in ART with the progestin (Dienogest) is a suitable substitute for GnRHa. Our findings have demonstrated a non-inferiority of the Dienogest in terms of ovarian response and pregnancy outcomes. Dienogest was better tolerated than GnRHa with a better tolerability score as assessed by FertiQoL score and a lower reported side effects rate. Furthermore, based on the two medications’ cost by the Egyptian pharmaceutical authority, the combined Dienogest pretreatment and ovarian stimulation regimen were significantly less expensive than the ultra-long protocol indicating better cost-effectiveness.

Our study was mainly powered to detect a difference in ovarian response in terms of the number of retrieved oocytes. In this respect, we could not detect any significant difference between the two intervention groups in both the quantitative as well as surrogate parameters of oocyte quality, i.e., the number of mature oocytes, the fertilization rate, and the number of transferrable embryos. Although our study was underpowered to detect a minimally significant difference in the clinical pregnancy rate, reassuringly, we could not detect any significant difference between the two intervention groups in pregnancy rates. This was the main limitation of the study. Therefore, it is considered mainly as a proof-of-concept study and a primer for further larger studies.

Another important aspect of the assessment of interventions is safety and tolerability and its impact on patients’ quality of life (QoL). We have, therefore, compared the ultra-long protocol with the Dienogest pretreatment for QoL outcomes. This has shown no difference in the patients in the two groups in their baseline QoL domains as assessed by the FertiQoL international (Arabic version) available from the Cardiff University website. This is a validated tool, which has been supported by professional organizations such as the European Society of Human Reproduction and Embryology (ESHRE) and the American Society of Reproductive Medicine (ASRM) for use in clinical practice and research. On assessing the treatment part of FertiQoL, Our study showed that the treatment tolerability scores for Dienogest are significantly better than the GnRHa group. This may not be surprising given the known hypo-estrogenic side effects of the GnRHa, which may substantially disrupt the patients’ QoL. This was further confirmed by the higher rate of side effects reported in the GnRHa group. This latter observation with the significantly higher cost of the ultra-long protocol may be essential factors that may lead to preference of the Dienogest over GnRHa if similar effects on ovarian response and pregnancy rates are further confirmed in future studies.

Recently, another RCT had shown that Dienogest pretreatment for patients with endometriosis before IVF was associated with a significantly lower number of oocytes retrieved and worse pregnancy outcomes [16]. Despite similarities in design, there are important differences between this trial and our study. First, the trial by Tamura et al. recruited patients with stage III-IV endometriosis, whereas our study was not restricted to a particular stage of endometriosis. It is known that patients with advanced-stage endometriosis, particularly with previous ovarian surgery, would have markedly compromised ovarian reserve. Second, in the trial by Tamura et al., patients were given a combined estrogen and progesterone treatment following the Dienogest suppression; the inclusion of estrogen could have counteracted the treatment effect of Dienogest. Furthermore, in that trial, GnRHa in the treatment arm was used in a flare protocol, which could also be counteracting the effect of Dienogest, whereas previous trials have shown that ultra-long protocol is more beneficial due to suppression of endometriosis.

## Conclusion

The findings of our study should be corroborated with larger adequately powered studies to confirm or refute a comparable effect of Dienogest to the ultra-long protocol on pregnancy outcomes, particularly impact on the live birth rate in endometriosis patients having ART. If confirmed, these findings would represent a major step towards the facilitation of endometriosis suppression before ART.

## Data Availability

The datasets used and/or analyzed during the current study available from the corresponding author on reasonable request.
